# Effect of Single Particle High-Speed Impingement on the Electrochemical Step Characteristics of a Stainless-Steel Surface

**DOI:** 10.3390/ma17123043

**Published:** 2024-06-20

**Authors:** Meihong Liu, Long Chai, Min Yang, Jiarui Cheng

**Affiliations:** 1 School-Run Factory (Engineering Training Center), Xi’an Aeronautical Institute, Xi’an 710077, China; liumeihong1223@163.com; 2 CCDC Changqing Downhole Technology Company, China National Petroleum Corporation, Xi’an 712042, China; cqjx_chail@cnpc.com.cn (L.C.); yang-min521@163.com (M.Y.); 3 Xi’an Key Laboratory of Wellbore Integrity Evaluation, Xi’an Shiyou University, Xi’an 710065, China

**Keywords:** erosion–corrosion, particle disturbance, electrochemical step, stainless steel repassivation

## Abstract

In the process of particle erosion and electrochemical corrosion interaction, the electrolyte flow state change, product film destruction, and matrix structure change caused by particle impact affect the electrochemical corrosion process. Such transient, complex physical and electrochemical changes are difficult to capture because of the short duration of action and the small collision area. The peak, step time, and recovery time in this transient step cycle can indirectly reflect the smoothness and reaction rate of the electrochemical reaction system, and thus characterize the resistance to scouring corrosion coupling damage of metals in liquid–solid two-phase flow. In this study, in order to obtain the electrochemical response at the moment of particle impact, electrochemical monitoring experiments using a specially designed miniature three-electrode system were used to test step-critical values, including step potential, current, and resistance, among others. Meanwhile, an electrochemical step model under particle impact considering boundary layer perturbation was developed. The experimental results reflect the effect law of particle impact velocity and particle size on the peak step and recovery period. Meanwhile, the effect of particle impingement on the electrochemical step of stainless steel in different electrolyte solutions was obtained by comparing the step curves in distilled water and Cl-containing water. The connection between the parameters in the electrochemical step model and in the particle impact, as well as the effect of the variation of these parameters on the surface repassivation process are discussed in this paper. By fitting and modeling the test curves, a new mathematical model of electrochemical step-decay under single-particle impact was obtained, which can be used to characterize the change pattern of electrochemical parameters on the metal surface before and after the impingement.

## 1. Introduction

Erosion–corrosion damage is influenced by multiple factors, including material properties, various hydrodynamic conditions, and the surrounding environment, making its study relatively complex [1]. The mechanism of damage caused by single-particle impacts in liquid–solid two-phase flow is a critical aspect of damage analysis. In practical conditions, erosion and corrosion occur simultaneously. The mechanical processes induced by single-particle erosion can lead to material deformation or detachment, while corrosion typically manifests as chemical or electrochemical reactions [2]. Additionally, the loss of pipe wall material occurs not only through the detachment of solid particles from the metal surface but also through dissolution in ionic form [3]. Current research on particle erosion and its impact on electrochemical reactions on metal surfaces encompasses many aspects, including corrosion current density, the influence of passive films, and impact damage models. These areas have received significant attention and in-depth study from numerous scholars.

Through experiments exploring the corrosion rate of electroplated copper–manganese materials in different media, Supriyatna et al. [4] concluded that higher applied current densities on steel significantly reduce its corrosion rate, with the corresponding electroplated layer thickness increasing with the current density. Additionally, Jan Mayén [5] discovered that the corrosion current density is also influenced by the tensile strength of the material, indicating a close relationship with its microstructural characteristics. Wang et al. [6] developed a novel non-destructive method for predicting corrosion current density in the presence of stray currents by integrating electrochemical laboratory measurements with data-driven techniques. Alhumade et al. [7] proposed an accurate corrosion current density model based on the adaptive neuro-fuzzy inference system (ANFIS) and experimentally validated the precision of the ANFIS model for corrosion current density.

In studies on the impact of passivation films, Li et al. [8] found that both repassivation and depassivation processes are related to the kinetic energy of solid particles, with depassivation delaying repassivation. They further theoretically predicted and experimentally validated the dependence of the critical flow velocity (CFV) for erosion–corrosion on solid particle concentration and diameter. Xu et al. [9] explored the fine structure of passivation films at the atomic scale, demonstrating the growth mode of the crystals. Ion channels within the passivation film explain the emergence of defect regions and the localized attack of chloride ions during corrosion. Hou et al. [10] discovered that introducing defects into the passivation film structure promotes corrosion degradation, experimentally demonstrating and revealing the fundamental deterioration process of passivation films. Wu et al. [11] studied the effect of high temperatures on the corrosion resistance of Fe-based amorphous coatings, finding that high temperatures accelerate the corrosion process and promote the thickening of the passivation film. Wang et al. [12] proposed a new method for studying repassivation time, which allows for comparison of the repassivation performance of different materials without the need for curve fitting or consideration of film growth mechanisms.

In the context of particle impact models, Khalifa [13] proposed a novel modeling approach for the agglomerate impact damage in wall-bounded particle turbulence based on artificial neural networks (ANN). This model incorporates the effects of shear factors, thereby enhancing its applicability. Mohammad et al. [14] investigated the formation of splat fragmentation during the flattening and solidification of droplets under plasma particle spraying conditions by establishing a numerical model. This study provided a kinetic perspective to explain the mechanisms affecting droplet flattening on solid surfaces. To predict the deformation of individual particles at different impact velocities, Schreiber [15] validated and optimized the Preston–Tonks–Wallace (PTW) model, which is effective in predicting the deformation of cold-sprayed particles. Ren [16] developed a semi-analytical model to estimate rock-breaking depth under stable damage conditions in particle jet impact, conducting numerical analyses on influencing factors. This model offers theoretical support for parameter optimization and field applications of particle jet impact drilling technology. In corrosive solutions, particle impacts on metal walls cause changes in near-wall fluid flow, leading to transient variations in electrochemical reaction parameters [17]. Changes in particle impact velocity, particle diameter, and particle count result in corresponding transient variations. Therefore, further research is needed to quantify the electrochemical responses induced by variations in particle parameters.

Based on the considerations above, this study investigates the effects of corrosive fluid flow rate and particle geometric parameters. The electrochemical response of pipe wall materials under single particle impact in chloride-containing solutions is examined, analyzing the interaction between mechanical and electrochemical damage. The results are used to explore the mechanisms by which particle impact influences electrochemical corrosion.

## 2. Experiment

In the pipe flow particle impact experiments, the low particle concentration in the liquid, combined with the small effective area of the working electrode in the micro three-electrode system, made sustained effective collisions difficult. Increasing the particle concentration further proved impractical for pumping and caused significant damage to the screw blades, pipes, and nozzles. Therefore, jet experiments were employed to study the electrochemical response of metal wall surfaces to particle impact.

**Figure 1:**
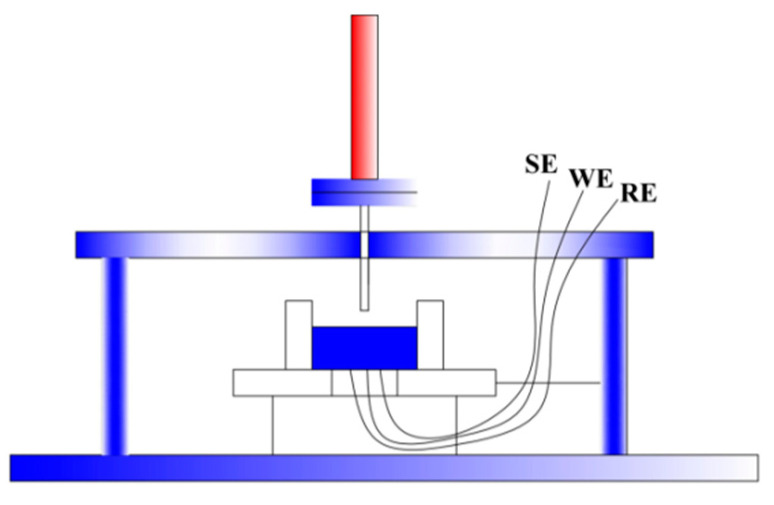
Diagram of particle impact experiment.

The particle impact experiments were conducted on the multiphase flow erosion–corrosion test rig [18], including the screw pump, liquid flowmeter, test chamber, sample holder, stirred tank in the liquid flow system and air compressor, buffer tank, filter drier, gas flowmeter, sand storage tank, and PLC feeder in the gas flow system. During the experiments, particles were added to the pipeline from the sand storage tank and carried by the liquid to the nozzle for ejection, impacting the target material surface (as shown in Figure 1). Once the particles collided with the sample surface, they were collected in the test section and the stirred tank because the sand would break after impact and affect the experimental results.

**Figure 2:**
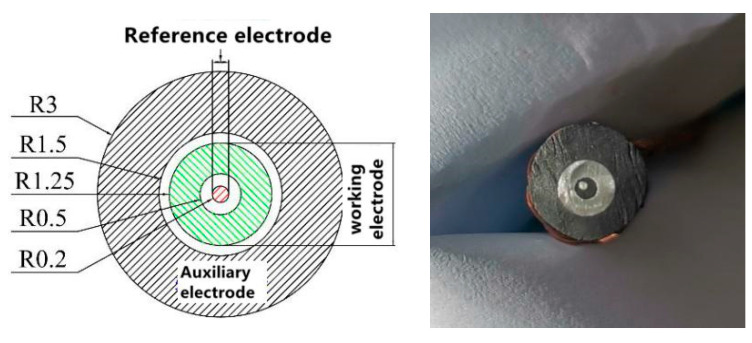
Diagram of three-electrode setup for particle impact electrochemical experiment.

The electrochemical experimental setup for particle impact is illustrated in Figure 2. An electrochemical workstation PARSTAT MC-1000 was used to test current changes (Ametek. Co., Ltd., Berwyn, PA, USA). An AgCl-saturated calomel electrode was employed as the reference electrode, and graphite served as the auxiliary electrode. The graphite electrode area was 20 times larger than the working electrode. The 304 stainless steel was used as the working electrode because its surface can be easily passivated. The saturated calomel reference electrode, which was placed in the test chamber, was connected to the standard three-electrode system. A long platinum wire was used as the counter electrode. For electrochemical monitoring, polarization curves were recorded by changing the electrode potential at a sweep rate of 0.2 mV/s.

**Figure 3:**
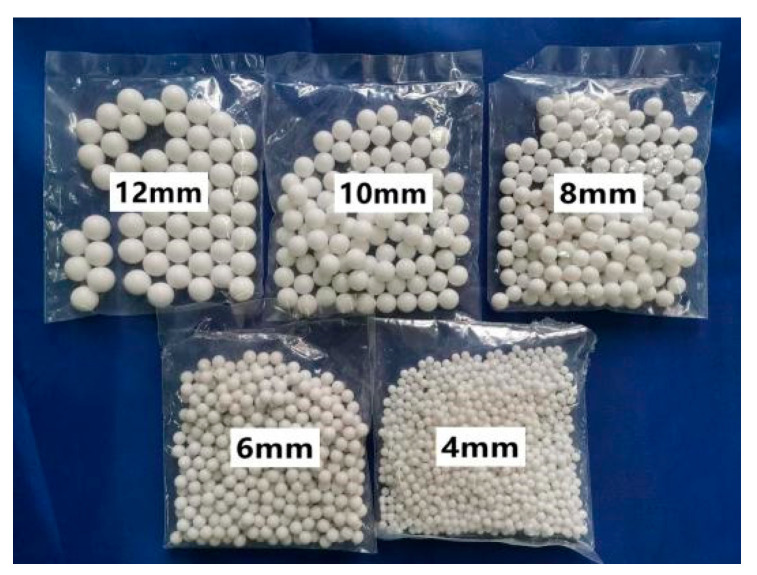
Single-particle impact experiment using plastic particles.

Two media were utilized in the experiments: H_2_O and H_2_O + 3.5 wt% NaCl. The jet nozzles had diameters of 10 mm and 15 mm, respectively. The particle impact angle was set to 90° to maximize the acceleration of reactant mass transfer. Single particle impact experiments utilized large-diameter spherical plastic particles made of methyl methacrylate (PMMA), characterized by their large diameter and low density. The large diameter maximizes disturbance of the mass transfer boundary layer and disrupts corrosion product films. The diameters used were 4 mm, 6 mm, 8 mm, 10 mm, and 12 mm, with a density of approximately 1200 kg/m^3^, as depicted in Figure 3.

Before initiating the experimental apparatus, the specimen was secured within the testing chamber, as depicted in Figure 1. The angle between the nozzle and the specimen was adjusted to 90°, with the nozzle aligned with the surface of the working electrode. The preparation of the solution in the mixing tank followed the same procedure as that for the fluid-induced corrosion experiment. Once the solution was prepared, mixing was initiated, the liquid circuit valve was opened, and the gas circuit valve was closed. The single-screw pump was then activated, and the flow rate was adjusted to the desired value. After stabilizing the flow rate for 10 min, the temperature and pressure inside the pipe were measured and recorded. Simultaneously, the electrochemical workstation was turned on for machine preheating. Once the flow rate in the experimental circuit stabilized, the three test channels of the electrochemical workstation were sequentially connected to the three electrodes for open-circuit potential and open-circuit current monitoring. The valve of the sand storage tank was then opened, allowing the liquid to carry the particles to impact the surface of the working electrode. The trend of potential and current changes during particle impact was observed. Upon completion of the experiment, the particles were collected within the testing chamber, the electrochemical workstation and pipeline instruments were shut off, and the connections to the three electrodes were disconnected.

## 3. Results

Due to the considerable size of the erosion–corrosion experimental rig, significant vibrations occur in the pipeline system at high flow rates, which can affect the monitoring results of potential and current. To minimize experimental errors, medium flow rates of 1 m/s, 2 m/s, 3 m/s, 4 m/s, and 5 m/s were selected for the single-particle impact experiments. For ease of analysis, the initial values of current and potential were uniformly set to 0 mV.

### 3.1. Results of Open-Circuit Potential Response

**Figure 4:**
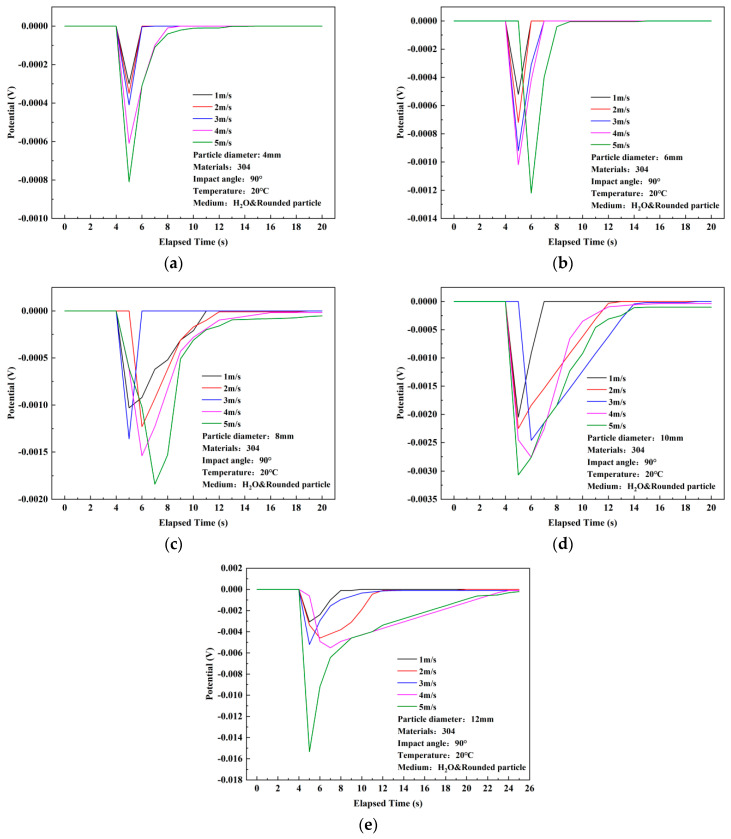
Experimental results of potential variation of 304 stainless steel under single particle impact in distilled water (flow rate comparison). (**a**) 4 mm, (**b**) 6 mm, (**c**) 8 mm, (**d**) 10 mm, (**e**) 12 mm.

**Figure 5:**
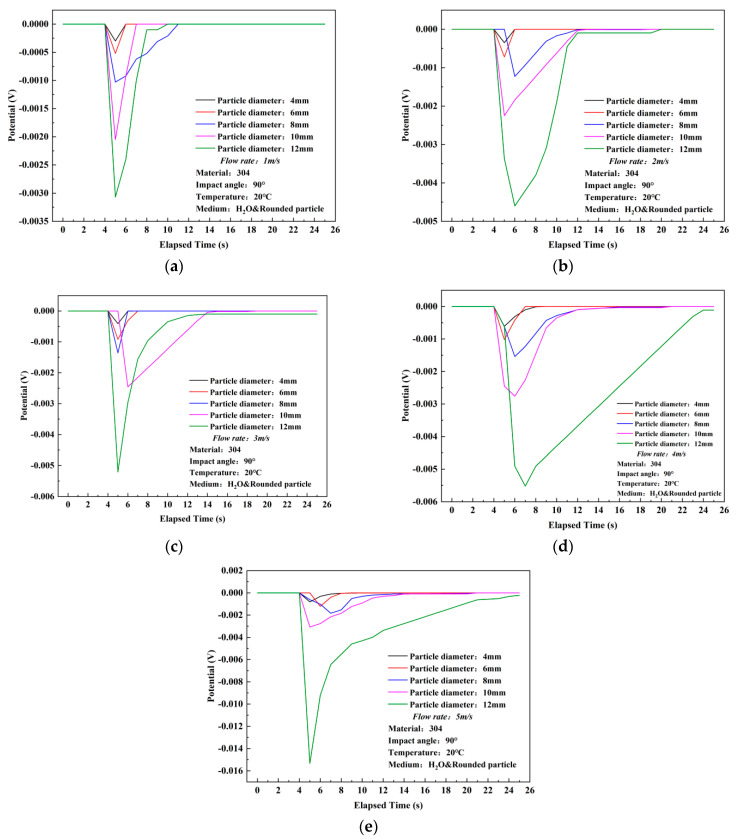
Experimental results of potential variation of 304 stainless steel under single particle impact in distilled water (comparison by particle diameter). (**a**) 1 m/s, (**b**) 2 m/s, (**c**) 3 m/s, (**d**) 4 m/s, (**e**) 5 m/s.

Differing from the current density exhibiting a peak value, the open-circuit potential (OCP) of the 304 stainless steel’s surface underwent a sudden negative shift after particle impact, displaying a minimum peak value. Similar to the trend observed in current density variations, the absolute value of the OCP peak increased with increasing flow velocity and particle diameter. Moreover, compared to the current density, the time required for the OCP to return to its initial value was longer, and the deviation between the recovery value and the initial value was greater, resulting in a negative shift in the final potential. In Figure 4, under different particle diameters, the OCP peak gradually increases with increasing flow velocity. For particle diameters of 4, 6, 8, and 10 mm, the absolute value of the potential exhibited relatively uniform increments. However, when the particle diameter increased to the maximum value of 12 mm, a sudden change in potential value occurred from 4 m/s (−0.00552 V) to 5 m/s (−0.01534 V), with the largest absolute value increment reaching 117.9%. Additionally, the test results indicate that with an increase in particle diameter, the absolute value of the peak potential exhibited an order of magnitude increase. The maximum potential peak values corresponding to particle diameters of 4 to 12 mm are −8.1 × 10^−4^ V, −0.00122 V, −0.00184 V, −0.00307 V, and −0.01534 V, respectively. In Figure 5, it is evident that the recovery time for the 12 mm diameter particles at various flow velocities is longer, with the longest recovery time observed at a flow velocity of 5 m/s and a particle diameter of 12 mm, reaching 20 s, indicating that the potential is more difficult to recover to its initial value compared to the current density.

### 3.2. Results of Surface Current Density Response

**Figure 6:**
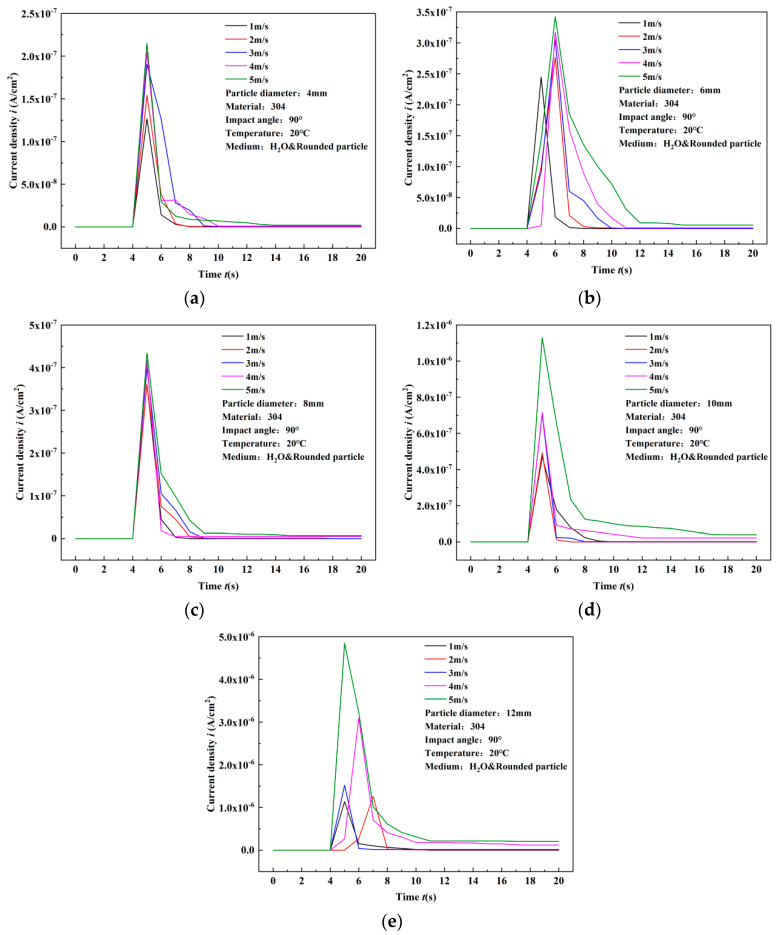
Experimental results of surface current density variation of 304 stainless steel subjected to single particle impact in water (comparison of flow velocities). (**a**) 4 mm, (**b**) 6 mm, (**c**) 8 mm, (**d**) 10 mm, (**e**) 12 mm.

**Figure 7:**
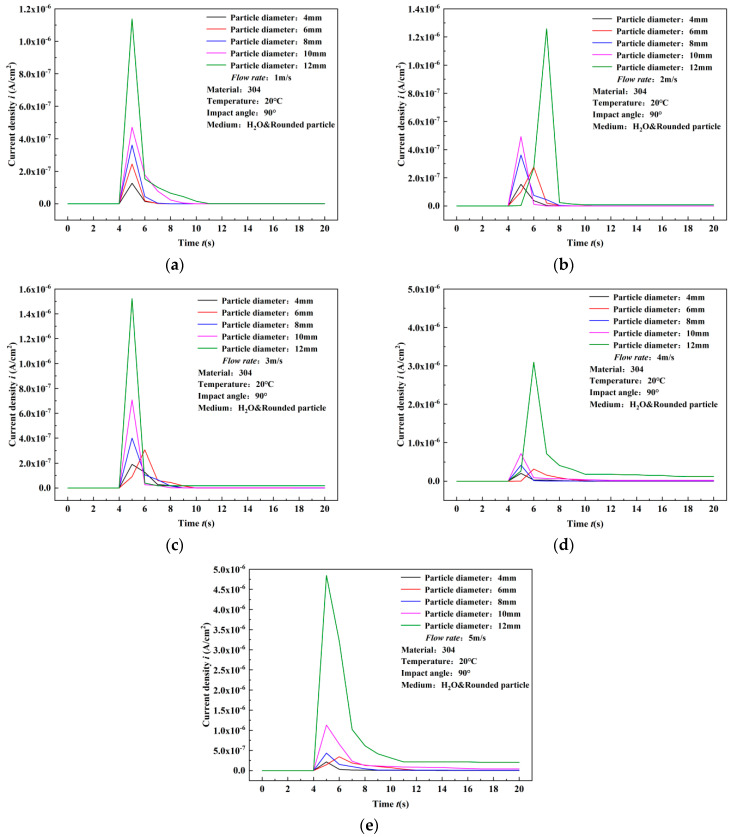
Experimental results of surface current density variation of 304 stainless steel subjected to single particle impact in water (comparison of particle diameters). (**a**) 1 m/s, (**b**) 2 m/s, (**c**) 3 m/s, (**d**) 4 m/s, (**e**) 5 m/s.

The experimental results of single particle impact on 304 stainless steel in water are shown in Figure 6. The transient peak value of surface current density of 304 stainless steel increased with the increase in flow velocity. Taking the example of the surface results under 4 mm particle diameter impact, the current density peak values at 1–5 m/s are 1.27 × 10^−7^ A/cm^2^, 1.54 × 10^−7^ A/cm^2^, 1.90 × 10^−7^ A/cm^2^, 2.04 × 10^−7^ A/cm^2^, and 2.15 × 10^−7^ A/cm^2^, respectively. With each 1 m/s increase in flow velocity, the percentage increase in current density peak values is 21.26%, 23.38%, 7.37%, and 5.39%. The maximum percentage increase in current density peak values occurs when the flow velocity increases from 2 m/s to 3 m/s, reaching 23.38%. This indicates that the increase in flow velocity significantly increases the peak value of surface current density caused by particle impact. Similar trends in changes in current density peak values under impact of particles of other diameters indicate a positive correlation between flow velocity and changes in current density peak values. Additionally, as shown in Figure 7, after being impacted by particles, the current density starts to recover from the peak value, but there is a deviation between the final recovery value and the initial value. This is because the particles cause damage to the surface of the material, resulting in deviation from the initial morphology after impact, and the material cannot self-repair to its original state. However, the deviation is small, indicating the good repassivation ability of 304 stainless steel after being impacted by particles in flowing water. At the same time, the step time of current density is short, while the recovery time of current density is longer, remaining within 6 s, which also reflects the good repassivation ability of 304 stainless steel.

Figure 7 illustrates the peak current density under different particle diameters at the same flow velocity. Comparative results show a noticeable increase in current density peak values with the enlargement of particle diameter at each flow velocity. When the flow velocity is 1 m/s, the current density peak values for particle diameters of 4, 6, 8, 10, and 12 mm are, respectively, 1.27 × 10^−7^ A/cm^2^, 2.45 × 10^−7^ A/cm^2^, 3.62 × 10^−7^ A/cm^2^, 4.71 × 10^−7^ A/cm^2^, and 1.14 × 10^−6^ A/cm^2^. With every 2 mm increase in particle diameter, the percentage increase in current density peak values is 92.91%, 47.76%, 30.11%, and 142.04%, respectively. The largest increase occurs between diameters 10 mm and 12 mm, representing an order of magnitude increase, indicating a nonlinear growth relationship between current density peak values and particle diameter. Similar trends in variations are observed under different flow conditions. This phenomenon is attributed to the cubic relationship between the diameter of spherical particles and their volume, which is proportional to the volume. Consequently, with constant particle density, the volume undergoes nonlinear changes, resulting in nonlinear changes in particle mass, and, subsequently, the impact energy of particles exhibits nonlinear amplification. Furthermore, with the increase in flow velocity, the time required for the surface current density peak value of 304 stainless steel to recover to its initial value also increases. Therefore, the variation in current density peak values with particle diameter becomes more significant. The highest increase occurs at a flow velocity of 5 m/s, where the current density corresponding to a diameter of 10 mm increases from 1.13 × 10^−6^ A/cm^2^ to 4.85 × 10^−6^ A/cm^2^ for a diameter of 12 mm, representing a percentage increase of 329.12%.

When 3.5 wt% NaCl is added to the experimental medium, the peak current density also increases with the increase in flow rate and particle diameter. Both the flow rate and particle diameter are positively correlated with the peak current density. Compared to the pure water medium, under the same flow rate and particle diameter conditions, the peak current density increases by orders of magnitude. In the pure water medium, the peak current density is in the range of 10^−6^ to 10^−7^, while in the 3.5 wt% NaCl medium, it is in the range of 10^−3^. Therefore, after particle impact, the deviation between the recovery value and the initial value of the current density is larger.

**Figure 8:**
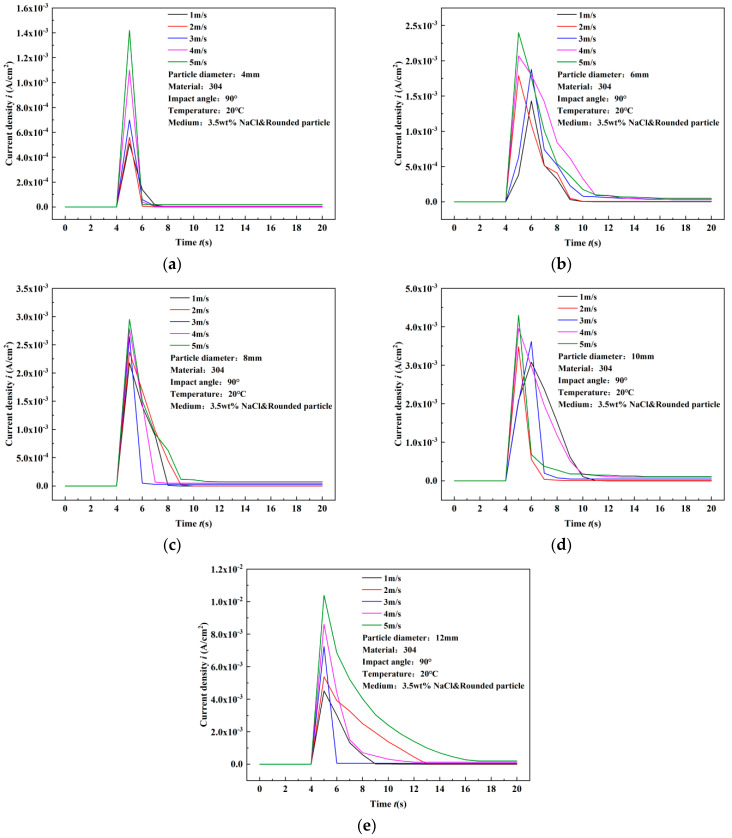
Experimental results of surface current density variation of 304 stainless steel under single particle impact in 3.5 wt% NaCl solution (comparison of flow rates). (**a**) 4 mm, (**b**) 6 mm, (**c**) 8 mm, (**d**) 10 mm, (**e**) 12 mm.

**Figure 9:**
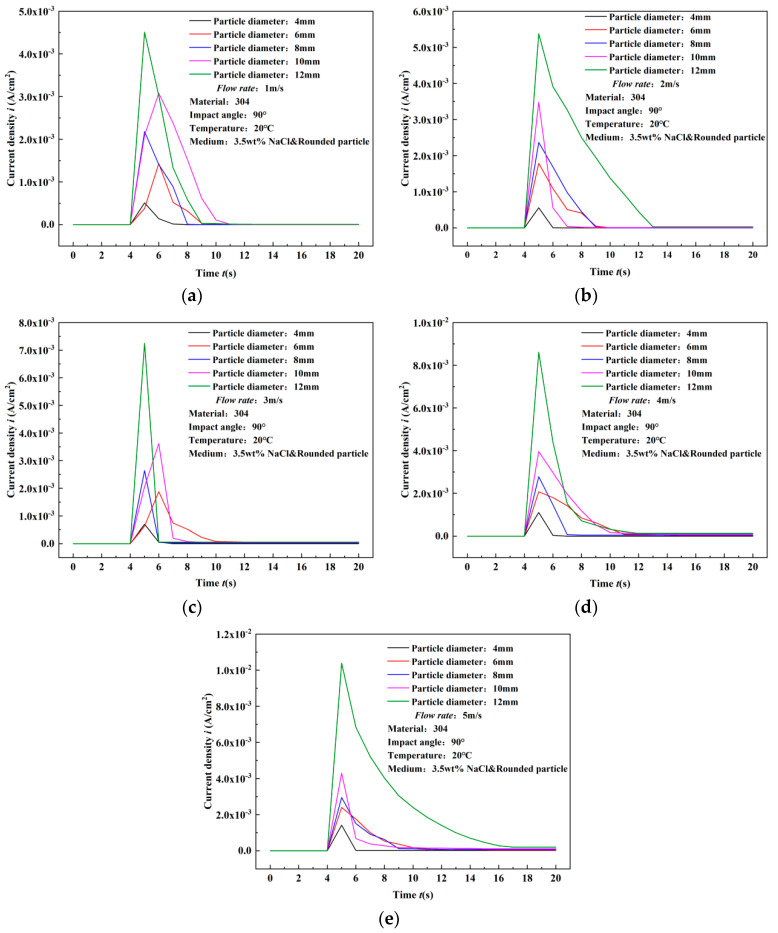
Experimental results of surface current density variation of 304 stainless steel under single particle impact in 3.5 wt% NaCl solution (comparison of particle diameters). (**a**) 1 m/s, (**b**) 2 m/s, (**c**) 3 m/s, (**d**) 4 m/s, (**e**) 5 m/s.

Due to the larger peak current density, the recovery time of the current density in the 3.5 wt% NaCl medium is longer. As shown in Figure 8e, when the 12 mm diameter particles impact the material surface at 4 m/s, the recovery time of the current density is 8 s, while at 5 m/s impact velocity, the recovery time reaches 12 s. It can be observed that the rising phase curve of the current density after particle impact also has a slope, rather than being vertical to the time axis, as shown in the typical response curve of Figure 9e corresponding to the 12 mm diameter particles.

For stainless steel, the transient increase in current density is attributed to passivation occurring on its surface. Due to the presence of a passivation film on the surface, the initial current density value is relatively low as the film impedes reaction processes. When large-diameter particles impact the surface of stainless steel, the passivation film ruptures, allowing active reactants to contact the metal substrate, intensifying the reaction process momentarily, thereby increasing the current density value. After reaching its peak, the current density diminishes with the repassivation process, gradually approaching the pre-impact initial value.

With increasing particle impact velocity, the peak current density also increases. Additionally, particles with greater mass (larger diameter) generate larger peak current density values. Since both impact velocity and particle diameter significantly influence the peak current density, this phenomenon is associated with particle kinetic energy. As reducing impact velocity or decreasing mass can diminish particle kinetic energy, there exists a kinetic energy threshold below which transient currents cannot be detected. The existence of this threshold is because there are no transient currents below the critical kinetic energy threshold. Upon impact on passivated surfaces, rapid transient current spikes do not occur because particle impacts below the threshold kinetic energy do not rupture the passivating oxide film on the metal surface.

## 4. Discussion

The impact of particles and electrochemical reaction corrosion exhibit a synergistic relationship with mutual influence. Particles disturb the mass transfer boundary layer, altering the mass transfer rate of reactants. Simultaneously, the impact dislodges the oxide film, carrying away reactants. Moreover, electrochemical reactions alter the surface structure of the metal, weakening local hardness. Concurrently, localized pitting occurs, disrupting the geometric organization structure.

In the experiment investigating the material’s current density response under single-particle impact, a positive step change was observed on the surface of the 304 stainless-steel electrode following particle impact [19]. As the diameter and impact velocity of the individual particles increased, the peak current density on the surface of the 304 stainless-steel electrode also increased. Concurrently, the time required for the current density to recover from its peak to near-initial values increased, indicating greater difficulty in maintaining system stability. The relatively short recovery time for 304 stainless steel suggests its robust self-repair capability. The results indicate a positive correlation between the particle impact-induced electrochemical corrosion rate and particle diameter, as well as impact velocity.

The surface open-circuit potential of the electrode undergoes a negative step change following particle impact, reaching a peak and gradually recovering with a nonlinear passivation trend. The open-circuit potential value after passivation recovery is lower than the potential value before particle impact, indicating that particle impact weakens the corrosion resistance of stainless steel in Cl^−^ medium. When particles impact the metal surface, they squeeze the near-wall flow mass transfer boundary layer, accelerating the reaction process, leading to a sudden positive step change in the current density and the appearance of a current density peak. As the particles move away from the surface, the current density gradually returns to near-initial values. Thus, based on the different response processes of current density, a model for the variation of current density on the electrode surface due to particle impact can be established, thereby predicting the material surface corrosion rate [20].

**Figure 10:**
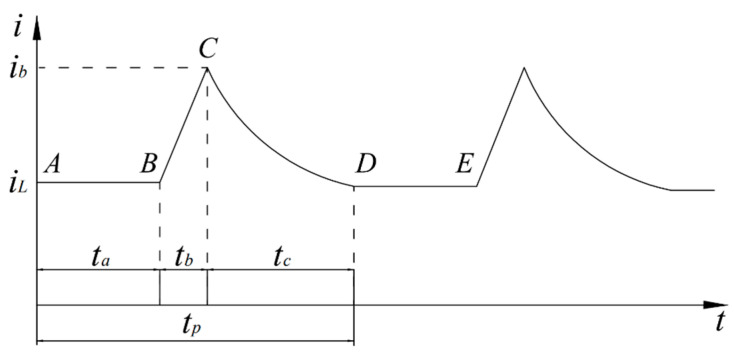
The schematic diagram illustrates the trend of current density variation caused by particle impact on the metal surface.

After metal is impacted by particles in a liquid–solid two-phase flow, the metal surface is exposed to the corrosive medium. The electrochemical reaction system on the surface experiences transient disturbances but eventually stabilizes. The electrochemical reaction current density is positively correlated with the corrosion rate. Following particle impact, the trend of current density variation is depicted in Figure 10.

Sections A–B represent pure liquid flow, where the surface undergoes a reaction equilibrium process, resulting in a stabilized current density. In sections B–C, the process involves the response of current due to particle impact. As a result of film rupture or sediment dispersion, the surface reaction current density reaches its maximum value, considered as the limit current density at point C. In sections C–D, following the departure of particles from the surface after impact, a transient process of recovery occurs, leading to the re-establishment of the surface electrochemical equilibrium system, ultimately reaching stability. Sections D–E are analogous to sections A–B, representing a stable process. For sections B–C, due to the lag in mass transfer and reaction, the actual time of current response lags behind the time of impact. However, because of the high impact velocity and short contact time with the surface, the lag in current change is neglected. After the disappearance of the impact effect in sections B–C, the reaction system re-establishes, initiating a repassivation process. The entire process of single particle impact on surface current density change can be segmented for calculation, thus further establishing a model for the current density response of single particle impact on the surface.

When particles enter the mass transfer boundary layer, they compress the boundary layer thickness, increase reactant concentration, and accelerate the mass transfer rate of the system. The near-wall viscous sublayer is characterized by laminar flow. After particles impact the viscous sublayer, the flow velocity changes from a laminar to a turbulent state. Therefore, Equation (1) is utilized to calculate the change in current density.

(1) iL=0.0189neFCaRe1/5ua1+0.6203Re−1/10Sc−1Lx

By altering the flow velocity within the conduit, the variation in the average flow velocity near the wall boundary layer at different velocities is computed. This provides a fitting function for the change in flow velocity and conduit diameter. By integrating the numerical changes in conduit diameter, the effect of particle impact on the convective growth current density is determined.


(2) $$i_{bl}\left(t\right)=A_{b}\cdot \lambda_{u}=i_{L}\cdot \left(0.0118u^{a}+0.1967d_{l}+0.2288\right)$$

By analyzing the convective, diffusive, and migratory current densities, as they are connected in parallel, the increase in current density due to particle impact on the metal wall is determined as follows:


(3) $$i_{b}\left(t\right)=i_{b1}\left(t\right)+i_{b2}\left(t\right)=i_{L}\cdot \left(0.0118u^{a}+0.1967d_{l}+0.2288\right)+\left(\sqrt{t_{b}}-1/\sqrt{t_{b}}\right)n_{e}FA_{e}D_{O}^{1/2}C^{a}$$

Upon completion of particle impact on the surface, firstly, there is a transition of the metal surface concentration from a small gradient to a large gradient, leading to a rapid decrease in the diffusion rate and a nonlinear decrease in the current density. Secondly, for stainless-steel surfaces, influenced by the passivation film coverage, the polarization resistance increases, resulting in a decrease in exchange current density. Through calculation, the time function of material diffusion current density after particle impact can be obtained:


(4) $$i_{c1}\left(t\right)=\frac{n_{e}FA_{e}D_{O}^{1/2}C_{O}^{a}}{\pi^{1/2}t_{c}^{1/2}\left(1+\kappa \varphi \right)}$$

For the single-particle impact process, the metal current density undergoes a full cycle variation, namely, impact–transient–recovery stages. Therefore, in calculating the corrosion rate, the current density–time function can be integrated to obtain the average value.

## 5. Conclusions

This paper begins with an experimental study on liquid–solid jet flow particle impact on a wall surface, focusing on the surface electrochemical response to individual particle impacts on 304 stainless steel. Subsequently, the process of a single-particle impact on the wall surface current density variation was calculated in stages, and a model for the response of the wall surface current density to single-particle impact was established. Based on the experimental and computational research conducted above, the following conclusions were drawn:

(1) Particle impact experiments indicate that there is a positive directional surge in current density on the surface of 304 stainless-steel electrodes after particle impact. With the gradual increase in the single particle’s diameter and impact velocity, the peak current density on the surface of 304 stainless-steel electrodes increases accordingly. Simultaneously, it takes longer for the current density to recover from its peak value to near its initial value, indicating that the system finds it increasingly difficult to maintain a stable state. The results demonstrate a positive correlation between the electrochemical corrosion rate of particle impact on the wall surface and the particle diameter and impact velocity.
(2) The open-circuit potential on the electrode surface undergoes a sudden negative deviation after being impacted by particles, reaching a peak value, and gradually recovers with a nonlinear passivation trend. The open-circuit potential value after passivation recovery is lower than the potential value before particle impact, indicating that particle impact weakens the corrosion resistance of stainless steel in Cl^-^ medium.
(3) When particles impact the metal surface, they squeeze the near-wall flow boundary layer, accelerating the reaction process, causing a sudden positive deviation in current density, leading to the appearance of a current density peak. As the particles move away from the wall, the current density gradually returns to near its initial value. Based on the different response processes of current density, a model for the variation of current density on the electrode surface due to particle impact was established to predict the corrosion rate of the material surface.

## Data Availability

The original contributions presented in the study are included in the article, further inquiries can be directed to the corresponding author.
